# Evaluation of clinical practice guidelines (CPG) on the management of female chronic pelvic pain (CPP) using the AGREE II instrument

**DOI:** 10.1007/s00192-021-04848-1

**Published:** 2021-06-19

**Authors:** Vishalli Ghai, Venkatesh Subramanian, Haider Jan, Jemina Loganathan, Stergios K. Doumouchtsis, CHORUS: An International Collaboration for Harmonising Outcomes, Research and Standards in Urogynaecology and Women’s Health (i-chorus.org)

**Affiliations:** 1 Department of Obstetrics and Gynaecology, Epsom & St Helier University Hospitals NHS Trust, Dorking Road, London, KT18 7EG UK; 2 St George’s University of London, Crammer Terrace, London, SW17 0RE UK; 3 Laboratory of Experimental Surgery and Surgical Research N.S. Christeas, National and Kapodistrian University of Athens, Medical School, Athens, Greece; 4 School of Medicine, American University of the Caribbean, Cupecoy, Sint Maarten

**Keywords:** AGREE II tool, Chronic pelvic pain, Guidelines, Sexual dysfunction, Systematic review

## Abstract

**Introduction and hypothesis:**

Variations in guidelines may result in differences in treatments and potentially poorer health-related outcomes. We aimed to systematically review and evaluate the quality of national and international guidelines and create an inventory of CPG recommendations on CPP.

**Methods:**

We searched EMBASE and MEDLINE databases from inception till August 2020 as well as websites of professional organizations and societies. We selected national and international CPGs reporting on the diagnosis and management of female CPP. We included six CPGs. Five researchers independently assessed the quality of included guidelines using the AGREE II tool and extracted recommendations.

**Results:**

Two hundred thirty-two recommendations were recorded and grouped into six categories: diagnosis, medical treatment, surgical management, behavioural interventions, complementary/alternative therapies and education/research. Thirty-nine (17.11%) recommendations were comparable including: a comprehensive pain history, a multi-disciplinary approach, attributing muscular dysfunction as a cause of CPP and an assessment of quality of life. Two guidelines acknowledged sexual dysfunction associated with CPP and recommended treatment with pelvic floor exercises and behavioural interventions. All guidelines recommended surgical management; however, there was no consensus regarding adhesiolysis, bilateral salpingo-oophorectomy during hysterectomy, neurectomy and laparoscopic uterosacral nerve ablation. Half of recommendations (106, 46.49%) were unreferenced or made in absence of good-quality evidence or supported by expert opinion. Based on the AGREE II assessment, two guidelines were graded as high quality and recommended without modifications (EAU and RCOG). Guidelines performed poorly in the “Applicability”, “Editorial Independence” and “Stakeholder Involvement” domains.

**Conclusion:**

Majority of guidelines were of moderate quality with significant variation in recommendations and quality of guideline development.

**Supplementary Information:**

The online version contains supplementary material available at 10.1007/s00192-021-04848-1.

## Introduction

Chronic pelvic pain (CPP) is a debilitating condition, affecting 15% of women worldwide [1]. It is associated with significant socio-economic burden and long-term morbidity [2]. CPP is defined as pain lasting > 6 months or recurrent episodes of abdominal/pelvic pain, hypersensitivity or discomfort often accompanied by elimination changes and sexual dysfunction [3]. CPP remains a challenging disorder to treat because of the complexities of pain sensation and unclear aetiology. Standard medical and surgical treatments seldom prove effective at improving quality of life and pain intensity among affected women [4]. Furthermore, the variation in outcome reporting of trials evaluating interventions has prevented the synthesis of data to identify effective treatments and draw clinically relevant conclusions in the context of guideline formation [5].

Clinical practice guidelines (CPG) are systematically developed statements using best available research evidence [6]. They aim to improve the delivery and quality of patient care and health outcomes. Adoption of CPG attempts to eliminate variation, standardize medical care and implement effective treatments. Guidelines are developed using standardized methods and processes including: engaging stakeholder groups, identifying, quality assessment and synthesis of research evidence as well using consensus methods to derive robust guideline recommendations. The methodological quality of guidelines has been inconsistent [7–10].

Guidelines based on poor evidence or those that fail to reflect the needs of women may contribute to the delivery of suboptimal, ineffective or even harmful interventions thereby compromising the quality of care. To date, there has been no evaluation of the methodological quality of national or international guidelines on female CPP. In this systematic review, we aimed to evaluate the methodological quality of CPP guidelines, produce a comprehensive inventory of recommendations and explore the relationships between recommendations and evidence.

## Methods

This systematic review was designed and reported according to the Preferred Reporting Items for Systematic Reviews and Meta-Analyses (PRISMA) guideline [11]. It was performed by a working group of CHORUS, an International Collaboration for Harmonizing Outcomes in Research and Standards in Urogynaecology and Women’s Health (https://i-chorus.org). This study is part of a wider project for establishing core outcome sets (COS) in CPP. We followed a methodological approach implemented successfully by previous studies by CHORUS working groups appraising the quality of clinical guidelines in various areas of gynaecology [7, 8, 10].

### Search strategy

A comprehensive literature search was undertaken using the MEDLINE and EMBASE databases. Searches were performed from database inception to August 2020 using the following MESH terms: “chronic pelvic pain”, pelvic pain”, “idiopathic chronic pelvic pain”, “guidance”, “guideline” and “recommendation”. Reference lists of included guidelines were hand-searched. We subsequently searched websites of specialist societies and professional organizations including gastroenterology, gynaecology, pain medicine and urology to identify additional guidelines. (A list of these can be found in Appendix 3.)

### Selection of guidelines

We included guidelines reporting on the diagnosis and management of CPP in women. Two researchers (VG, VS) reviewed the full text of retrieved guidelines independently to assess eligibility. Guidelines in languages other than English, specific to a particular condition (i.e. endometriosis, bladder pain syndrome), local/regional guidelines or if an updated guideline was available by the same organization were excluded. We excluded editorials, reviews, position statements, consensus statements, expert opinions, practice standards, practice alerts/bulletins and primary studies as these documents do not meet the criteria of assessment or purpose of the AGREE II tool [12]. Discrepancies regarding suitability for inclusion were resolved by discussion with a senior author (SKD) and a consensus reached. A PRISMA flow chart is included to demonstrate the search and guideline inclusion process (Appendix 2).

### Data extraction

Two researchers (VG and VS) extracted guidelines characteristics independently including: country of origin, year of publication, consensus methods, stakeholders involved, disease area examined, description of database search, search terms used, language restriction, date of searches, inclusion/exclusion criteria and quality assessment instrument.

Two researchers (VG and VS) mapped recommendations independently according to five pre-defined areas: (1) diagnosis, (2) medical management of pain, (3) surgical management of pain, (4) behavioural interventions, (5) complementary and alternative therapies for pain and (6) education and research. We defined behavioural interventions as exercise, dietary modification, physiotherapy and psychological treatment. Complementary/alternative therapies were defined as acupuncture, dry needling, homoeopathy, massage, reflexology and transcutaneous electric nerve stimulation (TENS). We reported levels of evidence used to support recommendations in guidelines. Supporting evidence was categorized according to the evidence-based medicine criteria [Cochrane reviews, systematic review, randomized control trials (RCT), non-randomized control trials, expert opinion and no reference]. Discrepancies were resolved by discussion with a third author (SKD). In cases where a recommendation supported by multiple sources of evidence of varying quality, we documented the highest quality of evidence.

### Quality assessment of included guidelines

Five researchers (VG, VS, HJ, JL, SKD) were trained and assessed the quality of guidelines independently using the AGREE II tool. This is a validated instrument consisting of 23 items grouped into six quality domains and two further global rating items: (1) scope and purpose (items 1–3), (2) stakeholder involvement (items 4–6), (3) rigour of development (items 7–14 items), (4) clarity of presentation (items 15–17), (5) applicability (items 18–21) and (6) editorial independence (items 22–23). Each item was rated using a seven-point Likert scale from 1 (strongly disagree) and 7 (strongly agree) [12]. The “Stakeholder Involvement” domain focuses on the extent to which the guideline was developed by the appropriate stakeholders and represents the views of its intended users [12]. The “Editorial Independence” is concerned with the formulation of recommendations not being unduly biased with competing interests [12]. Refer to Appendix 1 for a detailed description of each domain assessed by the AGREE II tool.

### Data analysis

Domain quality scores were calculated by the summation of scores per item and standardized using a prescribed equation [12]. An overall guideline score was derived as a mean of all six domain quality scores. This approach has been adopted by previous studies using the AGREED II tool to evaluate clinical guidelines [7, 13].

There is no consensus regarding using AGREE II scores to differentiate between high- and low-quality guidelines or recommendation of guidelines. For the purpose of this study, we considered a domain score < 50% as low quality [7, 10]. The overall guideline score was used to categorize guidelines into the following: low quality (0–30%), moderate quality (31–60%) and high quality (61–100%) [7]. Based on these ratings, high-quality guidelines were recommended, moderate quality guidelines were recommended with modifications and low quality guidelines were not recommended [9, 7].

Inter-rater reliability of assessments was tested for agreement using the Fleiss’ kappa co-efficient. Scores of ≤ 0 indicated no agreement, 0.01–0.20 indicated poor agreement, 0.21–0.40 indicated fair agreement, 0.41–0.60 indicated moderate agreement, 0.61–0.80 indicated substantial agreement and 0.81–1.0 indicated almost perfect agreement [14].

### Tabulation and data

Descriptive statistics were calculated for all domains (median, range and interquartile range). We used tables to map recommendations, the supporting evidence for recommendations and the hierarchy of evidence.

### Patient and public involvement

There has been no patient involvement as this study is a systematic review of existing research.

## Results

The electronic literature search yielded 1294 titles and abstracts. We screened 639 titles and abstracts following the exclusion of 655 duplicate records (Appendix 2 for PRISMA flow chart). A further three guidelines were identified from examination of references lists and websites of societies/associations (Appendix 3). In total, we included six guidelines comprising four national and two international guidelines: American College of Obstetricians and Gynaecologists (ACOG), American Society of Reproductive Medicine (ASRM), European Association of Urology (EAU), International Society of Psychosomatic Obstetrics and Gynaecology (ISPOG), Royal College of Obstetricians and Gynaecologists (RCOG) and The Society of Obstetricians and Gynaecologists of Canada (SOGC) (Table 1).

**Table 1:** Guideline characteristics

Guideline	Organization	Country/region of origin	Stakeholders (n; location)	Scope	Consensus method	Identification of evidence	Quality assessment of evidence
Consensus Guidelines for the Management of Chronic Pelvic Pain (2018)	The Society of Obstetricians and Gynaecologists of Canada (SOGC)	Canada	Obstetricians and Gynaecologists (12, various locations in Canada)	Diagnosis and management	Not reported	**Database:** Cochrane, MEDLINE **Search terms:** not reported **Language restriction:** not reported **Date of searches:** 1982–1994 **Inclusion/exclusion criteria:** unclear	Canadian Task Force om Preventative Health
International Society of Psychosomatic Obstetrics and Gynaecology (ISPOG) European Consensus Statement-Chronic Pelvic Pain in Women (2015)	International Society of Psychosomatic Obstetrics and Gynaecology (ISPOG)	Europe	Unclear	Medical, psychological and psychosomatic diagnostics and treatment	Unclear	**Database:** MEDLINE, PsychLit/PsychINFO, Annals of the German Society for Psychosomatic Gynaecology and Obstetrics **Search terms:** “chronic pelvic”, “endometriosis”, “pelvic congestion syndrome”, “bladder dysfunction”, “pelvic floor hypertonic disorder” **Language restriction:** not reported **Date of searches:** MEDLINE: 1966–December 2010), PsychLit/PsychINFO: November 2001–December 2007, Annals of the German Society for Psychosomatic Gynaecology and Obstetrics: 1983–2010 **Inclusion/exclusion criteria:** Unclear	Not reported
Consensus statement for the management of chronic pelvic pain and endometriosis: proceedings of an expert panel consensus (2002)	American Society of Reproductive Medicine (ASRM)	USA	Practicing gynaecologists (> 50, from various locations) Methodological experts (location or number not reported)	Medical and surgical care	Delphi	**Database:** MEDLINE **Search terms:** not reported **Language restriction:** not reported **Date of searches:** 1966–2001 **Inclusion/exclusion criteria:** Unclear	Not reported
The initial management of chronic pelvic pain (2012)	Royal College of Obstetricians and Gynaecologists (RCOG)	UK	Not reported	Investigation and management	Not reported	**Database:** Cochrane Library, Cochrane Register of Controlled Trials (CENTRAL), MEDLINE **Search terms:** “chronic disease”, “dysmenorrhoea”, “pelvic pain” **Language restriction:** not reported **Date of searches:** MEDLINE: 1966–2001 **Inclusion/exclusion criteria:** Unclear	Scottish Intercollegiate Guidelines Network (SIGN)
Guidelines on chronic pelvic pain (2014)	European Association of Urology (EAU)	Europe	Gynaecologist Neuro-Urologist Gastroenterologist, Urologists Pain medicine consultants Psychologist Sexologist (number or location not reported)	Management	Not reported	**Database:** Cochrane Library, Cochrane Register of Controlled Trials (CENTRAL), Bandolier, EMBASE, MEDLINE, PsychINFO **Search terms:** Not reported **Language restriction:** English **Date of searches:** January 1995–May 2011 **Inclusion/exclusion criteria:** Unclear	Oxford Centre for Evidence-based Medicine Levels of Evidence
Practice Bulletin. Chronic Pelvic Pain (2020)	American College of Obstetricians and Gynaecologists (ACOG)	USA	Obstetricians and Gynaecologists (number and location not reported)	Diagnosis and management	Not reported	**Database:** Cochrane, MEDLINE **Search terms**: not reported **Language restriction:** English **Date of searches:** January 2000–May 2019 **Inclusion/exclusion criteria: i**ncluded primary studies, commentaries, review articles, excluded abstracts	US Preventative Task Force

Although the ASRM guideline focused on women with endometriosis, it also considers the wider issue and common presenting complaint of female CPP. As our working group is developing a COS on CPP, the consensus of the authors was to include this guideline to provide a more comprehensive inventory of CPP regardless of diagnosis.

The ACOG practice bulletin, although it is not titled a CPG, was developed using methodology that met the criteria of assessment by the AGREE II tool and was therefore included.

The EAU guideline is directed to both women and men with CPP. For the purpose of this study we excluded recommendations that were relevant to men. However, this did not impact the assessment of guideline development as the AGREE II tool is an evaluation of the methods involved in the development of guidelines rather than the recommendations themselves.

### Guideline characteristics

Guidelines were published between 2002 and 2020. They specifically reported on the management including treatment of CPP. The number or type of stakeholders involved in the development of guidelines was not reported by two guidelines (ISPOG and RCOG). In four guidelines (ACOG, ASRM, EAU and SOGC), stakeholders included various health professionals (gynaecologists, urologists, neurologist, gastroenterologist, pain medicine, psychologist and sexologist) and methodological experts in clinical guideline development. The number of stakeholders in these guidelines ranged from 12 to 52. No guideline reported the involvement of women with CPP during the development process or reported their experiences of the recommended interventions. All guidelines developed recommendations applicable to high-income settings. One guideline (ASRM) described consensus development methods including the modified Delphi method. No guidelines provided a detailed search strategy used to identify supporting evidence for recommendations. Four guidelines (ACOG EAU, RCOG and SOGC) described methods to quality assess research evidence (Table 1).

### Methods on quality assessment of research evidence

Two guidelines were graded as high (EAU and RCOG) and four guidelines were graded as moderate quality (ACOG, ASRM, ISPOG and JOGC). The EAU and RCOG guidelines scored highly with overall mean scores of 69.86% and 62.50% respectively. The remaining four guidelines scored less than 50% overall mean scores including the ACOG (48.06%), ASRM (48.03%), SOGC (47.34%) and ISPOG (31.55%) guidelines (Table 2).

**Table 2:** AGREE II scores and recommendations

Guideline	Scope and purpose	Stakeholder involvement	Rigour of development	Clarity of presentation	Applicability	Editorial independence	Global rating	Fleiss kappa	Appraisers recommendation	Overall score
ACOG	77.78% (85)	34.44% (46)	49.17% (158)	81.11% (88)	0.83% (21)	45.00% (37)	66.67% (25)	0283 (CI 0.225–0.340)	Recommend with modifications	48.06%
ARSM	93.3% (99)	38.89% (50)	49.58% (159)	72.22% (80)	14.17% (37)	20.00% (22)	73.33% (27)	0.386 (CI 0.323–0.449)	Recommend with modifications	48.03%
EAU	82.22% (89)	60.00% (69)	62.50% (190)	97.78% (103)	25.00% (50)	91.67% (65)	80.00% (29)	0.243 (CI 0.154–0.332)	Recommend	69.86%
ISPOG	54.44% (64)	14.44% (28)	30.42% (113)	53.33% (63)	3.33% (24)	33.33% (30)	26.67% (13)	0.241 (0.180–0.301)	None	31.55%
SOGC	88.89% (95)	60.00% (69)	47.08% (153)	78.89% (86)	9.17% (31)	0.00 (10)	60.00% (23)	0.279 (CI 0.218–0.340)	Recommend with modifications	47.34%
RCOG	90.00% (96)	58.89% (68)	67.50% (202)	77.78% (85)	29.17% (55)	51.67% (41)	70.00% (26)	0.217 (CI 0.153–0.280)	Recommend	62.50%
Mean (SD)	81.11% (14.23)	44.44% (18.57)	51.04% (13.03)	76.85% (14.39)	13.61% (11.50)	40.28% (31.22)	NA	NA	NA	NA

Notes

() scores in brackets represent raw scores

% are scaled scores calculated using the following formula: (obtained score – minimum possible score)/(maximum score – minimum score)

Overall score calculated as mean of six domains

CI: confidence interval

SD: standard deviation

Mean domain scores varied greatly from 13.16% to 81.11%. Guidelines performed best in the “Scope and Purpose” (mean 81.11%, range 54.44–93.30) and “Clarity of Presentation” (mean 76.85%, range 53.33–97.78) domains. The ASRM and EAU guidelines achieved the highest scores in the “Scope and Purpose” domain. The ACOG and EAU guidelines achieved the highest scores in the “Clarity of Presentation” domain (Table 2). Guidelines were of low quality in the “Applicability” (mean 13.61%, range 0.83–29.17), “Editorial Independence” (mean 40.28%, range 0–91.67%) and “Stakeholder Involvement” (mean 44.44%, range 14.44–60.00) domains. The ACOG, SOGC and ISPOG guidelines recorded the lowest scores in these domains (Table 2).

The Fleiss kappa index varied between 0.217 to 0.386 and demonstrated fair agreement between reviewers (Table 2).

### Recommendations for clinical practice

In total, we extracted 228 recommendations across six guidelines. We grouped recommendations into the following categories: diagnosis (96 recommendations), medical treatment (72 recommendations), surgical treatment (29 recommendations), behavioural/physical interventions (18 recommendations), alternative treatments (7 recommendations) and education/research (5 recommendations).

Overall, 39 recommendations (17.11%) were comparable across guidelines including a comprehensive pain history, multifactorial nature of CPP including attributing muscular causes of CPP, an assessment of quality of life and multi-disciplinary approach. There was significant variation in recommendations regarding hormonal treatment, role of surgical interventions (adhesiolysis, hysterectomy, presacral neurectomy and uterosacral nerve ablation) as well the effectiveness of psychological and physical therapies.

Risks of interventions and procedures were reported in five guidelines (ACOG, ASRM, EAU, SOGC and RCOG). The rationale of clinical decision making was discussed in all guidelines,

### Diagnosis

Ninety-seven recommendations regarding diagnosis and investigation of CPP were made across guidelines. Of these, 39 recommendations (40.21%) were comparable across all six guidelines. Included guidelines described the multifactorial nature of CPP including causes such as bladder pain syndrome (BPS), irritable bowel syndrome (IBS) and muscular/myofascial dysfunction. All guidelines attributed CPP to muscular/myofascial causes including trigger points. Urological conditions such as BPS and interstitial cystitis were featured in five guidelines (ACOG, ASRM, EAU, ISPOG and RCOG). Three guidelines referred to endometriosis (ASRM, ISPOG and RCOG) and four guidelines alluded to nerve damage (ACOG, EAU, ISPOG and RCOG). IBS was described as a contributing factor by three guidelines (ACOG, ASRM and RCOG).

A detailed pain history was recommended by all guidelines; however, only two guidelines suggested a visual analogue scale (VAS) to measure pain intensity (ISPOG and SOGC). All guidelines recommended the evaluation of psychosocial factors including the recognition of concomitant mood disorders and assessment of quality of life. However, no guideline specified which patient-reported outcome measurement instrument to utilize.

Investigation of CPP varied among guidelines. The role of imaging such as transvaginal ultrasound to exclude adenomyosis, adnexal masses and endometriosis was recommended by three guidelines (ASRM, ISPOG and RCOG). MRI was recommended by the RCOG guideline to diagnose adenomyosis; however, its ability to accurately detect endometriotic deposits was uncertain (ASRM and RCOG). Diagnostic laparoscopy was recommended by five guidelines (ASRM, EAU, ISPOG, RCOG, SOGC). However, the RCOG guideline suggested laparoscopy as a second-line investigation if other therapeutic interventions fail. Cystoscopy was recommended by the SOGC guideline only.

A multi-disciplinary and multi-speciality approach to pain management was explicitly stated by five guidelines (ACOG, EAU, ISPOG, RCOG and SOGC). Furthermore, the ACOG, ISPOG and EAU guidelines also recommended that care of patients with CPP should be undertaken by pain medicine specialists.

### Medical treatment

Seventy-two recommendations were extracted regarding medical treatment of CPP. No recommendations were comparable in this domain. Nineteen recommendations focused on analgesia. Treatments with the highest grade of recommendations (i.e. level of evidence 1a and grade A recommendation) were paracetamol, NSAIDs (non-steroidal anti-inflammatory drugs), gabapentin, antidepressants, topical capsaicin and opioids (Table S3).

Simple analgesia including paracetamol and NSAIDs was specifically recommended by two guidelines (EAU and ASRM). The use of anticonvulsants such as pregabalin and gabapentin to treat CPP was described by four guidelines (ACOG, EAU, ISPOG and SOGC). Pain management with opioids was described by two guidelines (EAU and SOGC). However, only the EAU guideline stated which opioid to use, the preferred route of administration, a detailed consenting process including risks of addiction and dependency as well as plans for monitoring. In comparison, the ACOG guideline did not recommend treatment of CPP using opioids. Adjuvant medications such as tricyclic anti-depressants were suggested by four guidelines (ACOG, EAU, ISPOG and SOGC).

Hormonal treatment was recommended for underlying gynaecological causes such as endometriosis by three guidelines (ASRM, SOGC and RCOG). The use of oral contraceptives as a first-line treatment was stated by two guidelines (ASRM and SOGC). Progestins, Danazol and GnRH analogues were considered as first-line treatments in women with suspected endometriosis by the SOGC guideline. The ASRM guideline considered these as second-line treatments and recommended that an alternative diagnosis should be sought if adequate pain relief was not achieved. The RCOG also recommended hormonal treatment however did not specify which agents. A diagnostic laparoscopy was recommended in instances where pain was refractory to hormonal treatment after 3 to 6 months (Table S3).

Disease-specific treatments were described for BPS, chronic anal pain syndrome, irritable bowel syndrome (IBS) and myofascial dysfunction by four guidelines (ACOG, ASRM, EAU and SOGC). For suspected IBS, antispasmodics were recommended by one guideline (RCOG). A comprehensive list of BPS treatments including lifestyle modifications, oral medications, neuromodulation, intravesical therapies including trigonal and submucosal injections as well as hydrodistension was detailed by one guideline (EAU). Management of chronic anal pain syndrome using inhaled salbutamol, botulinum toxin A, electrogalvanic stimulation and percutaneous tibial nerve stimulation was described by the EAU guideline (Table S3).

### Surgical treatment

This domain consisted of 29 recommendations. However, no recommendations were comparable despite surgical management described by all guidelines. Twenty-four recommendations specifically referred to surgical procedures. The highest grade of recommendations supported hysterectomy for refractory symptoms, hysterectomy with ovarian conservation for endometriosis/adenomyosis and treatment of peritoneal pockets frequently associated with endometriosis and did not support routine adhesiolysis (Table S4). It must be noted that recommendations were graded A or B and the level of evidence varied between I to II-2. One guideline did not describe a level of evidence supporting recommendations (ACOG). No recommendation of the highest grade (i.e. level of evidence I and grade A) was noted in the surgical domain (Table S4).

Hysterectomy was recommended by three guidelines for refractory and severe symptoms (ASRM, ISPOG and SOGC). One guideline advised bilateral salpingo-oophorectomy during hysterectomy to relieve symptoms such as CPP secondary to endometriosis (ASRM). In contrast, the SOGC guideline suggested ovarian conservation was an acceptable option during hysterectomy for adenomyosis/endometriosis. The use of HRT after hysterectomy and bilateral oophorectomy for CPP secondary to endometriosis was described by a single guideline (SOGC).

Presacral neurectomy and laparoscopic uterosacral nerve ablation were discussed by two guidelines (ASRM and SOGC). The ASRM guideline did not recommend these in instances of endometriosis-related CPP. However, it did suggest that presacral neurectomy may be beneficial in reducing the severity of dysmenorrhoea. The SOGC guideline stated the role of presacral neurectomy for pain reduction in endometriosis remained unclear (Table S4).

There was a difference in recommendations regarding adhesiolysis. Laparoscopic adhesiolysis was supported by the ISPOG guideline; however, the SOGC and ACOG guidelines did not recommend routine laparoscopic adhesiolysis in the context of CPP. The RCOG guideline recognized the benefit of dividing dense vascular adhesions to reduce pelvic pain but did not support adhesiolysis of fine adhesions (Table S4).

Three guidelines (ASRM, ISPOG and SOGC) recommended surgical treatment of endometriosis using laparoscopic ablation or excision. A single guideline (EAU) described sacral neurostimulation for chronic anal pain syndrome. Transurethral resection of bladder lesions in type 3 BPS was recommended by the EAU guideline. Ablative surgery for BPS was advised by a single guideline as a last resort and by knowledgeable surgeons only (EAU) (Table S4).

### Behavioural/physical interventions

Eighteen recommendations described behavioural interventions. No recommendations were comparable across guidelines. The highest grade (i.e. level of evidence I and grade A) of recommendations supported psychological interventions combined with medical/surgical, biofeedback treatment in pelvic pain and dyssynergic defecation and biofeedback as an adjunct to muscle exercises in overactive pelvic floor muscles (Table S5).

Psychological treatments were recommended by three guidelines (ACOG, EAU and ISPOG). The EAU and ISPOG guidelines recommended integrating psychological interventions with standard medical/surgical treatments for CPP. The EAU guideline specifically referred to the role of psychological treatment in various conditions including PBS, urethral pain syndrome and chronic vulvar pain.

The ACOG guideline recommended cognitive behavioural therapy, pelvic floor physiotherapy or sexual therapy alone or in combination to manage myofascial dysfunction and dyspareunia secondary to CPP. Only one guideline (EAU) recommended training pelvic floor muscles to improve quality of life and sexual function. The EAU guideline advocated using behavioural strategies for patients and their partners with sexual dysfunction secondary to CPP.

Physiotherapy was recommended by a single guideline (EAU) for BPS and pelvic floor overactivity. Pelvic floor muscle treatment was considered a first-line treatment in CPP by the EAU guideline.

Only one guideline (SOGC) reported the use of exercise in the treatment of CPP due to myofascial dysfunction. No further details regarding the type of exercise (aerobic or resistance) were provided (Table S5).

### Complementary and alternative treatments

Seven recommendations focused on alternative treatments. No recommendations were comparable across guidelines. The highest grade (i.e. level of evidence I and grade A) of recommendations supported treatment of myofascial trigger points by dry needling or pressure (Table S5). There were conflicting statements regarding dry needling of trigger points (EAU and ISPOG).

One guideline reported limited data supporting the use of alternative therapies to treat CPP including acupuncture, trigger point treatment, reflexology, biofeedback, distension therapy, homoeopathy and Thiele massage (ISPOG). A single guideline (EAU) supported the use of alternative therapies such as TENS to treat CPP. There were no consistent recommendations regarding acupuncture and use was limited to specific clinical scenarios. The EAU guideline did not recommend acupuncture in the treatment of BPS. However, the ACOG guideline supported use of acupuncture in CPP secondary to muscoskeletal aetiology (Table S5).

### Recommendations for education and research

Five recommendations were relevant to education and research. No recommendations were comparable in this domain. One guideline advised incorporating CPP into the curricula of health professionals (SOGC). Two guidelines outlined future research priorities including the role of gene therapy, effectiveness of surgical management and investigating myofascial and sexual dysfunction in CPP (EAU and SOGC).

### Evidence supporting recommendations

Three guidelines (EAU, RCOG and SOGC) reported the grading of evidence used to support recommendations. Each guideline used a different grading scale including the Oxford Centre for Evidence-based Medicine Levels of Evidence (EAU), the Scottish Intercollegiate Guidelines Network (SIGN) grading system (RCOG) and the Canadian Task Force on Periodic Health Exam grading (SOGC). Of note, the ACOG guideline evaluated evidence using the US Preventative Services Task Force grading system; however, the level of evidence supporting each recommendation was not reported.

The total number of references cited in guidelines ranged from 22 to 644. The number of Cochrane reviews cited by each guideline ranged from 1 to 20. One guideline did not use any Cochrane reviews to support recommendations. The total number of RCTs used per guideline ranged from 6 to 32. Almost half of the recommendations (106, 46.49%) were unreferenced, made in the absence of good quality evidence or supported by expert opinion.

## Discussion

### Main findings

There is significant variation in CPP guideline quality and recommendations. We identified 189 unique recommendations but only 39 recommendations were comparable across 6 guidelines. A lack of consensus was observed in recommendations regarding medical and surgical treatments as well as complementary/alternative therapies. Nearly half of recommendations (108, 46.55%) were unreferenced, or made in absence of good quality evidence or supported by expert opinion. The quality of guidelines was variable; only two guidelines were assessed as high quality using the AGREE II instrument. Guidelines performed poorly in the “Applicability”, “Editorial Independence” and “Stakeholder Involvement” domains.

### Strength and limitations

To our knowledge, this is the first study to systematically appraise the methodological quality and map recommendations of CPP guidelines. We used robust and reproducible methods that have been successfully implemented in previous studies. To improve the scientific rigour of this review, five reviewers trained in using the AGREE II tool assessed guidelines, thereby minimizing possible bias arising from data collection or inherent limitations of the AGREE II tool related to inter-observer variations. Nevertheless, the inter-rater agreement among reviewers was fair.

This study has limitations. The AGREE II instrument is used to assess the rigour of guideline development rather than the quality of guideline content. Scoring achieved using the AGREED II tool is not a reflection of applicability or implementation in clinical practice [15]. All reviewers were from the same medical speciality and this may have influenced scores assigned to guidelines. However, as our focus was female CPP we feel that the clinical and research expertise of the assessors may counteract this limitation. Our research group included individuals with clinical expertise in minimal access gynaecological surgery (HJ and VG), reproductive medicine and surgery (VS) and urogynaecology (JL and SKD), thus providing different clinical perspectives from sub-specialities within gynaecology. Our ability to synthesize and compare recommendations was limited by the varied scope and small number of guidelines. However, by including guidelines that are diverse may provide a comprehensive and informative overview of available guidance. We included guidelines published between 2002 and 2018. However, only two guidelines (ACOG and SOGC) were published after the introduction of standardized terminology in CPP syndromes [3]. The use of standardized terminology can help improve identification, diagnosis and treatment. Our understanding and approach to CPP is an evolving process; however, guidelines may not be an accurate reflection if they are not updated regularly.

### Interpretation

This systematic review reflects variations in guideline recommendations and the poor quality of guideline development. Similar results were found in studies critically appraising the quality of guidelines including BPS, endometriosis, obstetric perineal lacerations and the use of transvaginal mesh implants in prolapse [7, 8, 10, 16]. Challenges of guideline development are not unique to a specific area of medicine but represent a generic issue arising from a lack of standardization. Our findings contribute to the existing body of evidence supporting the need to harmonize national and international guidelines. A coordinated and collaborative approach is required among guideline developers. Guidelines should be developed using transparent and robust methods such as those outlined by the AGREE II tool. This will facilitate the comparability and harmonization of guidelines and their recommendations as differences in guideline development methods can result in varying recommendations. Standardized guideline development will minimize any unwarranted and unjustified variations in clinical practice.

Various terminology has been used by authors and organizations to describe clinical practice recommendations. These have included clinical practice guidelines, consensus statements, position statements, practice alerts and hybrid terms such as consensus guidelines. Such terms can be indicative of the level of evidence and strength of recommendations. However, authors have used these terms interchangeably with a lack of consistency and available evidence. This poses a particular problem and possibly a challenge in harmonization of clinical guidance, as encountered in this study, of identifying “true” clinical practice guidelines that are developed using rigorous methodology. The use of inconsistent terminology may mislead clinicians about the level of confidence to place in recommendations and the process by which they were developed. Further consensus initiatives are needed for transparency and clarity regarding the definitions of these terminologies [17].

Guidelines support and provide an evidence base to the clinical decisions made in daily practice. However, we observed that almost half of all recommendations were made despite the absence of good quality evidence. The shortage of primary research supporting the management of women with CPP may prohibit the development of useful guidelines [18]. Furthermore, the quality of existing RCTs evaluating CPP interventions is variable with significant variability in outcome selection and reporting [5]. A working group within CHORUS is currently in the progress of establishing core outcome sets (COS) in CPP. Implementation of a COS will promote greater reporting consistency and reduce outcome reporting bias by stipulating a minimum set of criteria to report. It will also facilitate the comparability and synthesis in meta-analysis to produce high-quality results leading to informed healthcare decisions.

Diagnostic and therapeutic guideline recommendations are helpful in standardizing and improving the quality of care. However, they are limited and may not be relevant or apply in every clinical field of practice. CPP is a manifestation resulting from various underlying conditions that may evolve and develop into regional pain disorders. The complex aetiology of CPP and scarcity of available evidence may cause clinicians to resort to their own experience or seek expert opinions. A holistic approach is needed as underlying causes, treatment options and concerns of women with CPP can vary. Additionally, groups/societies publish guidelines for their members rather than a broader audience. The focus and scope of guidelines may be influenced by the clinical/scientific theme, interests or priorities of publishing societies/professional groups. For example, professional bodies for gynaecologists may focus on endometriosis only in women presenting with CPP. This may have contributed to the variability of recommendations for the management of female CPP observed in this study. The inclusion of a multidisciplinary stakeholder committee will help produce concise and collaborative recommendations that prevent duplicate investigations and the recommendation of ineffective treatments. This study indicated that guidelines performed poorly in the Stakeholder Involvement domain reflecting unilateralism in their approach to CPP including a lack of engagement and participation with their target audience, i.e. women with CPP.

In this review, all guidelines recommended surgical intervention; however, there was no consensus among guidelines. The lack of consistent recommendations presents difficulty when supporting or refuting the effectiveness of surgical procedures, such as adhesiolysis, to manage female CPP. The benefit of bilateral salpingo-oophorectomy during hysterectomy was also unclear with contradictory advice given in two guidelines. There is a place for hysterectomy in the management of CPP particularly due to endometriosis; however, patient selection is paramount [19]. Furthermore, recent studies have shown that bilateral removal of normal ovaries during hysterectomy does not result in improved outcomes but in fact is associated with increased morbidity [19–21]. Guidelines should not only focus on identifying effective interventions by utilizing the best available evidence but also provide guidance on which patient factors may contribute to the success and failure of such interventions [22].

In an era of patient-centred care, it was concerning that no guideline reported including women with CPP in the development process. Views and perceptions of women with CPP can be incorporated to frame the overarching theme of future guidelines as well as ensure the quality and relevance of recommendations. For example, all guidelines discussed the association of sexual abuse and CPP. However, no guideline specifically outlined recommendations pertaining to sexual abuse. Furthermore, no guideline reported women’s experiences to support the use of recommended interventions. These insights are invaluable for policymakers and guideline developers. The identification of barriers and facilitators can influence the successful implementation of interventions. In this review, included guidelines may not have incorporated qualitative research findings because of a lack of primary qualitative studies exploring the experiences of women with CPP [18, 23].

The detrimental impact of CPP on quality of life outcomes has been described and identified as a priority for women with CPP [18]. Despite this, quality of life assessment is only reported by half of RCTs evaluating treatments for CPP [5]. Our findings demonstrated that all guidelines referred to the measurement of quality of life; however, there was no recommendation regarding which measurement instrument to utilize. Our previous systematic review identified 17 quality of life measurement instruments; however, further research is required to assess the validity of such instruments in a CPP population [5].

There is an increased prevalence of sexual dysfunction observed in women with CPP compared with those without CPP (69.6% versus 30.4%) [24]. Underlying pelvic floor dysfunction including pelvic floor muscle overactivity and myofascial trigger points have been implicated in sexual disorders. Despite the impact of sexual dysfunction on the psychological and emotional well-being of women, we only identified two guidelines that recognized concurrent sexual dysfunction in women with CPP [25]. The ACOG and EAU guideline recommended pelvic floor physiotherapy to improve quality of life and sexual function. Although there is increasing evidence to support the use of physiotherapy in CPP and sexual dysfunction, it remains underused in clinical practice [26].

Similarly, exercise has been successfully incorporated into multidisciplinary treatment programmes to treat female CPP [4, 27]. Available evidence suggests that physical activity/exercise as an intervention is associated with few adverse events and may improve pain intensity, physical function and consequently quality of life [28]. However, our study demonstrated that only one guideline recommended the use of exercise in the management of CPP despite such benefits. Guideline developers need to incorporate multidisciplinary treatment modalities such as physiotherapy and exercise to maximize treatment benefits derived from conventional medical/surgical interventions.

Our findings are consistent with other studies that noted the Applicability, Editorial Independence and Stakeholder Involvement domains as areas of improvement for future guideline development [7, 8, 10, 16].

Our findings suggest that guidelines performed poorly in the Stakeholder Involvement domain. Guidelines failed to engage and involve women with CPP in the development process. Most guidelines were unsuccessful at including individuals from relevant professional groups therefore narrowing their scope to managing CPP. For example, only a single guideline included health professionals from various disciplines such as gastroenterology, neurology, pain medicine, urology and psychology. The inclusion of a multidisciplinary guideline development group will help produce concise and collaborative recommendations. Furthermore, in complex conditions such as CPP which may be due to a single or multiple concurrent causes or indeed idiopathic, input from multiple specialities is required. It is vital that clinical pathways are efficient and coordinated. A diagnostic pathway can often be a frustrating and negative experience for women with CPP hindered by delay [18]. Guideline developers such as the National Institute of Clinical Excellence (NICE) have initiatives such as the Patient Public Involvement Programme (PPIP) to support opportunities for patients and the public to be involved in developing guidance. Additionally, guideline developers can contact patient organizations to assist with the recruitment and inclusion of patients on their panels.

The Applicability domain refers to the implementation of guideline recommendations. Various factors can influence the successful implementation of guidelines such as the awareness of recommendations, associated costs and resource implications. Development of guidelines without effective and structured implementation strategies may not lead to the expected changes in clinical practice. Identification of barriers and facilitators in advance and incorporating these into the guideline development process can lead to tailored implementation strategies. These can improve adherence and avoid unnecessary investigations and inadequate interventions [29].

Maintaining editorial independence is vital to ensure the reliability and validity of recommendations. Influence or interference from funding bodies or conflicts of interest from members of the guideline development group can introduce bias and undermine the credibility of the guideline development process. Conflicts of interest can be mitigated by guideline developers obtaining and presenting full and transparent disclosures.

## Conclusion

The majority of guidelines were of moderate quality with significant variation in recommendations and the quality of guideline development. Adoption of standardized guideline development methods will ensure guideline recommendations are relevant, reliable and transferrable to clinical practice.

### Supplementary Information

ESM 1 (DOCX 34 kb)

ESM 2 (DOCX 21 kb)

ESM 3 (DOCX 19 kb)

## Figures and Tables

**Fig. 1:**
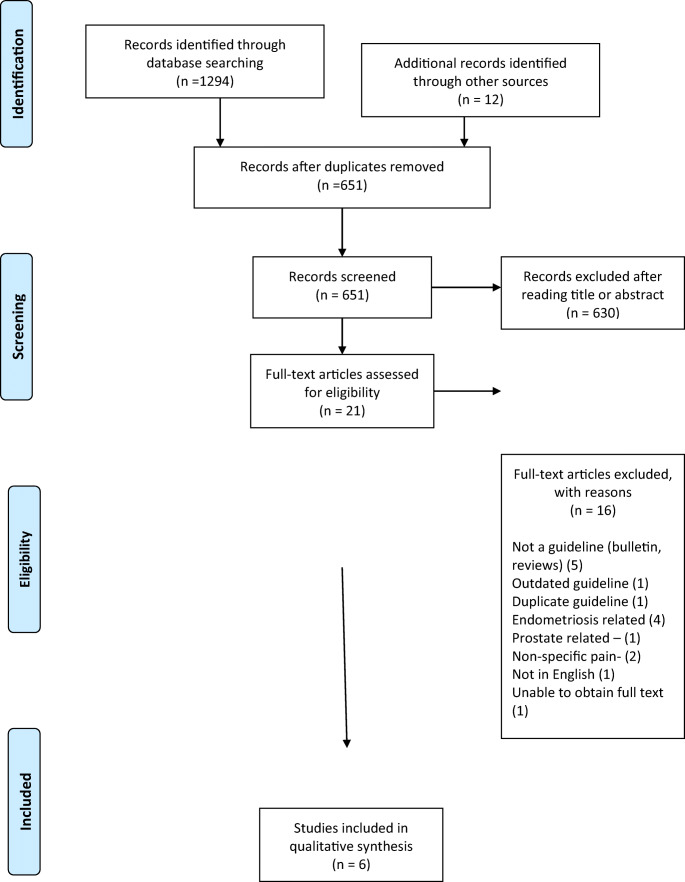
PRISMA flow diagram

## Appendix 1

**Table 3:** AGREE II domains and definitions

Domain	Definition
1. Scope and Purpose	Is concerned with the overall aim of the guideline, the specific health questions and the target population
2. Stakeholder Involvement	Focuses on extent to which guideline was developed by appropriate stakeholders and represents the views of its intended users
3. Rigour of Development	Relates to the process used to gather and synthesize the evidence, the methods to formulate the recommendations and to update them
4. Clarity of Presentation	Deals with the language, structure and format of the guideline
5. Applicability	Pertains to the likely barriers and facilitators to implementation, strategies to improve uptake and resource implications of applying the guideline
6. Editorial Independence	Is the formulation of recommendations not being unduly biased with competing interests

## Appendix 3. List of specialist society websites searched

American Association of Family Practice (AAFP).

American College of Obstetricians and Gynaecologists (ACOG).

British Association of Urological Surgeons (BAUS).

British Fertility Society (BFS).

British Society of Gastroenterology (BSG).

British Society of Gynaecological Imaging (BSGI).

British Society of Gynaecological Endoscopy (BSGE).

British Society of Pain (BPS).

British Society Psychosomatic Obstetrics, Gynaecology and Andrology (BSPOGA).

British Society of Urogynaecology (BSUG).

British Society of Biopsychosocial Obstetrics and Gynaecology.

Institute of Psychosexual Medicine.

eGuidelines.co.uk

European Association of Urology (EAU).

European Society of Gastroenterology (ESGE).

European Society of Gynaecology.

European Society Gynaecological Endoscopy (ESGE).

Faculty of Pain Medicine.

Indian College of Obstetricians and Gynaecologists (ICOG).

International Association for the study of pain.

International Federation of Gynaecology and Obstetrics (FIGO).

International Urogynecology Association (IUGA).

National Institute of Clinical Excellence (NICE).

Royal Australian and New Zealand College of Obstetrics and Gynaecology (RANZOG).

Royal College of Obstetricians and Gynaecologists (RCOG).

Scottish Intercollegiate Guidelines Network (SIGN).

Turning Research into Practice.
