# Supplementary material for: Evaluation of clinical practice guidelines (CPG) on the management of female chronic pelvic pain (CPP) using the AGREE II instrument
Source: Int Urogynecol J. 2021 Jun 19;32(11):2899–912. doi: 10.1007/s00192-021-04848-1 (PMC8536555; doi:10.1007/s00192-021-04848-1)
Supplement: Supplementary file 1 — (DOCX 34 kb) [file 192_2021_4848_MOESM1_ESM.docx]

**Table S3. Medical Treatment of Chronic Pelvic Pain with levels of evidence (LoE) and grades of recommendation (GoR)**

| **Guidelines** | **ACOG ^(i)^** | **ASRM** | **EAU ^(ii)^** | **ISPOG** | **RCOG ^(iii)^** | **SOGC ^(iv)^** |
| --- | --- | --- | --- | --- | --- | --- |
| **Generic – Analgesia** |  |  |  |  |  |  |
| Analgesics should not be used for CPP over a prolonged period. |  |  |  | **RCT** |  |  |
| Offered appropriate analgesia to control pain even if no other therapeutic manoeuvres are yet to be initiated. If pain is not adequately controlled, consideration should be given to referral to a pain management team or a specialist pelvic pain clinic |  |  |  |  | **No LoE**  **Good practice point** |  |
| Paracetamol for somatic pain |  |  | **LoE 1a**  **GR A** |  |  |  |
| NSAIDs for pain with inflammatory process |  |  | **LoE 1a**  **GR A** |  |  |  |
| Anti-depressants for neuropathic pain | **No LoE reported GR B** |  | **LoE 1a**  **GR A** | **RCT** |  | **LoE II-3**  **GR B**  **(and antibiotics)** |
| Gabapentin for neuropathic pain | **No LoE reported**  **GR B** |  | **LoE 1A**  **GR A** |  |  |  |
| Gabapentin for women with CPP |  |  | **LoE 2b**  **GR B** |  |  |  |
| Topical capsaicin for neuropathic pain |  |  | **LoE 1a**  **GR A** |  |  |  |
| Opioid therapy can be considered for pain control in CPP under adequate supervision |  |  | **No evidence** |  |  | **LoE II-3**  **GR B** |
| Opioids can be used for chronic non-malignant pain |  |  | **LoE 1a**  **GR A** |  |  |  |
| Opioids are not recommended for the treatment of  chronic pelvic pain. Patients already on opioids  should be slowly weaned. | **LoE not reported**  **GR B** |  |  |  |  |  |
| All other reasonable treatments must have been tried and failed before using opioids for chronic non-urogenital pain |  |  | **No evidence** |  |  |  |
| Where there is a history or suspicion of drug abuse, a psychiatrist or psychologist with an interest in pain management and drug addiction should be involved. (chronic non-urogenital pain) |  |  | **No evidence** |  |  |  |
| The patient should undergo a trial of opioids in chronic non-urogenital pain |  |  | **No evidence** |  |  |  |
| The dose required needs to be calculated by careful titration in chronic non-urogenital pain |  |  | **No evidence** |  |  |  |
| The patient should be made aware (and possibly give written consent): (indication chronic non-urogenital pain) • Opioids are strong drugs and associated with addiction and dependency. • Opioids will normally only be prescribed from one source (preferably the family doctor). • The drugs will be prescribed for fixed periods of time and a new prescription will not be available until the end of that period. • The patient may be subjected to spot urine and possibly blood checks to ensure that the drug is being taken as prescribed, and that non-prescribed drugs are not being taken. • Inappropriate aggressive behaviour associated with demanding the drug will not be accepted. • Hospital specialist review will normally occur at least once a year. • The patient may be requested to attend a psychiatric/psychological review. Failure to comply with the above may result in the patient being referred to a drug dependency agency and the use of therapeutic, analgesic opioids being stopped. |  |  | **No evidence** |  |  |  |
| Morphine is the first-line opioid, unless there are contraindications to morphine or special indications for another drug. (chronic non-urogenital pain) • The drug should be prescribed in a slow-release/modified-release form. • Short-acting preparations are undesirable and should be avoided where possible. • Parenteral dosing is undesirable and should be avoided where possible. |  |  | **No evidence** |  |  |  |
| **Chronic pelvic pain due to suspected endometriosis/gynaecological cause -First line treatment** |  |  |  |  |  |  |
| First line: NSAIDs or oral contraceptives or both |  | **No evidence** |  |  |  |  |
| First line: NSAIDs should be used around tine of menses with cyclical pain or continuous pain |  | **No evidence** |  |  |  |  |
| First line: oral contraceptives, progestins, Danazol and GnRH agonists |  |  |  |  |  | **LoE I - and II-I**  **GR A** |
| Hormonal therapy for 3-6 months before offered a diagnostic laparoscopy |  |  |  |  | **LoE 1+ to 4**  **GR B** |  |
| **Chronic pelvic pain due to suspected endometriosis – second line treatment** |  |  |  |  |  |  |
| Second line: If first line medical therapy fails to relieve pain symptoms, then trial with advanced medical therapy with Danazol, GnRH agonists, progestin such as Medroxyprogesterone. Alternatively, a laparoscopy can be offered |  | **No evidence** |  |  |  |  |
| Second-line treatment with a two month trial of Danazol, GnRH agonist or progestin such as medroxyprogesterone and continue for 6 months or longer |  | **No evidence** |  |  |  |  |
| If adequate pain relief not obtained from second line treatment (Danazol, GNRH, progestin such as medroxyprogesterone), then consider an alternative diagnosis |  | **No evidence** |  |  |  |  |
| Second line treatment is recommended instead of surgery in those women with CPP that have not responded to NSAIDs or OC /or form whom they are contraindicated |  | **No evidence** |  |  |  |  |
| **Chronic pelvic pain due to suspected endometriosis – surveillance and maintenance** |  |  |  |  |  |  |
| If adequate pain relief is obtained from the selected agent, then an appropriate maintenance management regimen should be initiated, keeping in mind that pain often has multiple causes and may recur, requiring re-evaluation and treatment revisions. |  | **No evidence** |  |  |  |  |
| Women who obtain adequate relief from first line treatment (NSAIDs or oral contraceptives) but for whom symptoms return upon completion or cessation of medical treatment should be maintained on the therapeutic regimen that previously produced relief. |  | **No evidence** |  |  |  |  |
| Maintenance regime with NSAIDs or oral contraceptive or both |  | **No evidence** |  |  |  |  |
| Women who obtain relief from second line medical treatment but for whom symptoms return upon reversion to NSAIDs or oral contraceptives should be considered for long-term treatment with second line medical treatment. |  | **No evidence** |  |  |  |  |
| Women who obtain relief from laparoscopic resection and/or ablation should be considered for continuation treatment with NSAIDs or oral contraceptives or with advanced medical therapy if symptoms return. |  | **No evidence** |  |  |  |  |
| Post-surgical treatment for endometriosis, maintenance adjuvant medical treatment with Danazol, GnRH agonist, progestin like medroxyprogesterone |  | **No evidence** |  |  |  |  |
| Post-surgical treatment for endometriosis, no evidence for oral contraceptive |  | **No evidence** |  |  |  |  |
| **Non-invasive treatments** |  |  |  |  |  |  |
| Nerve blocks for CPP part of broad management plan |  |  | **LoE 3**  **GR C** |  |  |  |
| Neuromodulation for pelvic pain, role with increasing research |  |  | **LoE 3**  **GR C** |  |  |  |
| **Irritable Bowel Syndrome** |  |  |  |  |  |  |
| Treatment with anti-spasmodic agent |  |  |  |  | **LE 1++/4**  **GR A** |  |
| **Myofascial Dysfunction** |  |  |  |  |  |  |
| Trigger point injections of saline, anesthetic, steroids, or opioids, in isolation or in combination with other treatment modalities, are recommended to improve pain and functional ability in patients with myofascial CPP | **LoE not reported**  **GR B** |  |  |  |  |  |
| **Chronic Anal Pain Syndrome** |  |  |  |  |  |  |
| Inhaled salbutamol should be considered in the intermittent chronic anal pain syndrome. |  |  | **LoE 3**  **GR C** |  |  |  |
| Botulinum toxin A and electrogalvanic stimulation can be considered in the chronic anal pain syndrome |  |  | **LoE 1b**  **GR B** |  |  |  |
| Percutaneous tibial nerve stimulation can be considered. |  |  | **LoE 1b**  **GR B** |  |  |  |
| **Bladder Pain Syndrome** |  |  |  |  |  |  |
| Offer subtype and phenotype-oriented therapy |  |  | **LoE 4**  **GR A** |  |  |  |
| Corticosteroids are not recommended for long-term treatment |  |  | **LoE 3**  **GR C** |  |  |  |
| Offer hydroxyzine for the treatment of BPS. |  |  | **LoE 1b**  **GR A** |  |  |  |
| Consider cimetidine as a valid oral option before invasive treatments for BPS |  |  | **LoE 2b**  **GR B** |  |  |  |
| Administer amitriptyline for use in BPS |  |  | **LoE 1b**  **GR A** |  |  |  |
| Offer oral pentosanpolysulphate sodium for the treatment of BPS. |  |  | **LoE 1a**  **GR A** |  |  |  |
| Treatment with oral pentosanpolysulphate sodium plus subcutaneous heparin is recommended especially in low responders to pentosanpolysulphate sodium alone. |  |  | **LoE 1b**  **GR A** |  |  |  |
| Antibiotics can be offered for BPS when infection is present or highly suspected. |  |  | **LoE 2b**  **GR C** |  |  |  |
| Prostaglandins are not recommended. |  |  | **LoE 3**  **GR C** |  |  |  |
| Cyclosporin A might be used in BPS but adverse effects are significant and should be carefully considered. |  |  | **LoE 1b**  **GR B** |  |  |  |
| Duloxetine is not recommended |  |  | **LoE 2b**  **GR C** |  |  |  |
| Oxybutynin might be considered |  |  | **LoE 3**  **GR C** |  |  |  |
| Gabapentin might be considered for oral treatment. |  |  | **LoE 3**  **GR C** |  |  |  |
| Opioids might be used in BPS in disease flare-ups. Long-term application only if all treatments have failed. |  |  | **LoE 2b**  **GR C** |  |  |  |
| Administer intravesical lidocaine plus sodium bicarbonate prior to more invasive methods for BPS |  |  | **LoE 1b**  **GR A** |  |  |  |
| Administer intravesical pentosanpolysulphate sodium before more invasive treatment alone or combined with oral pentosanpolysulphate sodium. |  |  | **LoE 1b**  **GR A** |  |  |  |
| Consider intravesical heparin before more invasive measures alone or in combination treatment for BPS. |  |  | **LoE 3**  **GR C** |  |  |  |
| Consider intravesical hyaluronic acid before more invasive measures for BPS. |  |  | **LoE 2b**  **GR B** |  |  |  |
| Consider intravesical chondroitin sulphate before more invasive measures. |  |  | **LoE 2b**  **GR B** |  |  |  |
| Administer intravesical DMSO before more invasive measures |  |  | **LoE 1b**  **GR A** |  |  |  |
| Consider intravesical bladder wall and trigonal injection of BTX-A if intravesical instillation therapies have failed. |  |  | **LoE 3**  **GR C** |  |  |  |
| Administer submucosal injection of BTX-A plus hydrodistension if intravesical instillation therapies have failed. |  |  | **LoE 1b**  **GR A** |  |  |  |
| Intravesical therapy with BCG is not recommended. |  |  | **LoE 1b**  **GR A** |  |  |  |
| Intravesical therapy with clorpactin is not recommended. |  |  | **LoE 3**  **GR A** |  |  |  |
| Intravesical therapy with vanilloids is not recommended |  |  | **LoE 1b**  **GR C** |  |  |  |
| Bladder distension is not recommended as a treatment of BPS. |  |  | **LoE 3**  **GR C** |  |  |  |
| Electromotive drug administration might be considered before more invasive measures |  |  | **LoE 3**  **GR C** |  |  |  |
| Neuromodulation might be considered before more invasive interventions for BPS. |  |  | **LoE 3**  **GR B** |  |  |  |

**Abbreviations**

ACOG; American College of Obstetricians and Gynecologists, ASRM; American Society of Reproductive Medicine, BPS; Bladder Pain Syndrome, BSO; Bilateral salpingo-oophorectomy, CPP; Chronic Pelvic Pain, EAU; European Association of Urology, ISPOG; International Society of Psychosomatic Obstetrics and Gynecology, RCOG; Royal College of Obstetricians and Gynaecologists, SOGC; The Society of Obstetricians and Gynaecologists of Canada

**Note**

1. American College of Obstetricians and Gynecologists (ACOG) uses A-C to grade recommendations, “A based on good and consistent scientific evidence, “B” based on limited or inconsistent scientific evidence, “C” based on consensus and expert opinion. Level of evidence were reported as “I” if evidence obtained from at least one properly designed RCT, “”II-1” if evidence from well-designed controlled trials without randomisation, “II-2” if evidence from well-designed cohort or case-control studies, “II-3” if evidence from comparisons between times or places with or without the intervention, “III” opinions of respected authorities, based on clinical experience, descriptive studies, or reports or expert committees
2. European Urology Association (EUA) uses A-C to grade recommendations, “A” based on evidence from at least one RCT, “B” recommendation based on evidence from well-conducted clinical studies but without randomisation, “C” recommendation made despite the absence of clinical studies. Level of evidence described as ‘1a” evidence from meta-analysis of RCTs, “1b” evidence from at least one RCT, “2a” evidence from well-designed controlled study without randomisation, “2b” evidence from at least one other type of well-designed quasi-experimental study, “3” evidence from well-designed non-experimental studies, such as comparative studies, correlation studies and case-reports, “4” evidence from expert committee reports or opinions or clinical experience or respected authorities.
3. Royal College of Obstetricians and Gynaecologists (RCOG) uses A-D, ‘A’ as high grade of recommendation and ‘D’ as very low and “Good Practice Point” and rated as “A” : ‘1++’ if at least one meta-analysis, systematic review or randomised clinical trial, and as “1+” if a systematic review of randomised controlled trials or a body of evidence principally of “1+” studies, “B”: as body of evidence including studies rated as “2++” if systematic reviews of case control or cohort studies or cohort studies with a low risk of bias/confounding factors or extrapolated evidence from studies as “1++’” or “1+”, ‘C’: as body of evidence including studies rated as “2+” if well-designed case-control or cohort study or extrapolated evidence from studies as “2++” and “D”: as evidence level 3 if case reports or case-series or 4, if expert opinion or extrapolated evidence from studies as “2+”.
4. Society of Obstetricians and Gynaecologists of Canada (SOGC) uses A-E to grade recommendations, “A” suggests that there is good evidence to support the recommendation for a diagnostic test/ intervention /treatment and “E” as good evidence not to support the recommendation for a diagnostic test/ intervention /treatment. Level of evidence were reported as “I” if evidence obtained from at least one properly designed RCT, “”II-1” if evidence from well-designed controlled trials without randomisation, “II-2” if evidence from well-designed cohort or case-control studies, “II-3” if evidence from comparisons between times or places with or without the intervention, “III” opinions of respected authorities, based on clinical experience, descriptive studies, or reports or expert committees
