# Supplementary material for: Evaluation of clinical practice guidelines (CPG) on the management of female chronic pelvic pain (CPP) using the AGREE II instrument
Source: Int Urogynecol J. 2021 Jun 19;32(11):2899–912. doi: 10.1007/s00192-021-04848-1 (PMC8536555; doi:10.1007/s00192-021-04848-1)
Supplement: Supplementary file 2 — (DOCX 21 kb) [file 192_2021_4848_MOESM2_ESM.docx]

**Table S4. Surgical Treatment of Chronic Pelvic Pain with levels of evidence (LoE) and grades of recommendation (GoR)**

| **Guideline** | **ACOG^(i)^** | **ASRM** | **EAU ^(ii)^** | **ISPOG** | **RCOG ^(iii)^** | **SOGC ^(iv)^** |
| --- | --- | --- | --- | --- | --- | --- |
| **Adhesiolysis** |  |  |  |  |  |  |
| The routine use of laparoscopic adhesiolysis is not recommended  for the management of chronic pelvic pain. | **GR A** |  |  |  |  |  |
| Current evidence does not support routine adhesiolysis for chronic pelvic pain. However, diagnostic laparoscopy remains of value |  |  |  |  |  | **LoE I**  **GR B** |
| There is no evidence to support the division of fine adhesions in women with chronic pelvic pain. |  |  |  |  | **LoE 1+ Good practice point** |  |
| Division of dense vascular adhesion should be considered as this is associated with pain relief. |  |  |  |  | **LoE 1+ Good practice point** |  |
| Laparoscopy is recommended as a preferred procedure for the surgical treatment of findings (adhesiolysis) in female CPP. |  |  |  | **RCT** |  |  |
| **Hysterectomy** |  |  |  |  |  |  |
| Hysterectomy alone without BSO has little or no place in the management of women who have CPP secondary to endometriosis alone. |  | **No evidence** |  |  |  |  |
| Hysterectomy for endometriosis or adenomyosis with ovarian conservation can be an acceptable alternative. The patient should be informed of the possible consequences (residual ovary syndrome, persistent pain, and reactivation of endometriosis |  |  |  |  |  | **LoE II-2**  **GR A** |
| Hysterectomy can be indicated in the presence of severe symptoms with failure of other treatment when fertility is no longer desired. |  |  |  |  |  | **LoE I**  **GR B** |
| For CPP patients, hysterectomy is a final intervention. It should only be performed if there is a manifest organic pathology. |  |  |  | **No evidence** |  |  |
| BSO, with or without hysterectomy, should be reserved for women who have completed their child bearing and who realize the potential impact of castration on other health parameters such as risk of osteoporosis, sexual dysfunction, and other menopausal issues. |  | **No evidence** |  |  |  |  |
| **Ovarian cystectomy** |  |  |  |  |  |  |
| Ovarian cystectomy, rather than oophorectomy, should be an individual decision, based on the patient's age and wishes, fertility issues, and surgical feasibility |  |  |  |  |  | **LoE II-3**  **GR B** |
| The management of adnexal torsion should be determined according to the patient's age and wishes, fertility issues, and surgical judgment. |  |  |  |  |  | **LoE II-3**  **GR B** |
| **Laparoscopic uterosacral nerve ablation (LUNA) and neurectomy** |  |  |  |  |  |  |
| LUNA and presacral neurectomy should not be routine methods in the treatment of CPP. |  |  |  | **RCT** |  |  |
| Patients with dysmenorrhea who have not responded to medical therapy may be offered Pre Sacral Neurectomy at laparotomy or, if the operator is adequately experienced, via laparoscopy. |  | **No evidence** |  |  |  |  |
| Further delineation of the role of appendectomy and of presacral neurectomy appears warranted in the management of endometriosis-related pain |  |  |  |  |  | **LoE III**  **GR A** |
| Available evidence suggests that LUNA does not benefit women with CPP associated with endometriosis. |  | **No evidence** |  |  |  |  |
| Neuromodulation and neurolysis must be considered experimental procedures in the treatment of CPP. |  |  |  | **No evidence** |  |  |
| **Endometriosis** |  |  |  |  |  |  |
| If patients with CPP undergo diagnostic laparoscopy and endometriosis is identified and thought to cause or contribute to the pain, conservative treatment with laparoscopically directed excision, ablation, or both should be strongly considered provided that the operator is adequately experienced. |  | **No evidence** |  |  |  |  |
| If endometriosis has been confirmed as a finding in the CPP patient, the surgical (laparoscopic) removal of the implants is seen as the therapy of choice (depending on the situation, it may be followed by postoperative drug treatment). |  |  |  | **No evidence** |  |  |
| Pelvic peritoneal defects (pockets) are frequently associated with endometriosis and should be treated surgically |  |  |  |  |  | **LoE II**  **GR B** |
| The location and/or extent of disease in combination with the patient’s desires regarding future fertility are important considerations. |  | **No evidence** |  |  |  |  |
| **Hormone replacement therapy (HRT) after hysterectomy** |  |  |  |  |  |  |
| In women with an intact uterus, when total hysterectomy has not been performed because of technical difficulties, the recurrence of endometriosis contraindicates the use of HRT. |  |  |  |  |  | **LoE I**  **GR B** |
| Since the rate of recurrence of endometriosis with HRT in women undergoing hysterectomy plus BSO is very low, HRT should not be contraindicated |  |  |  |  |  | **LoE I**  **GR B** |
| **Bladder Pain Syndrome** |  |  |  |  |  |  |
| Transurethral resection (or coagulation or laser) of bladder lesions, in BPS type 3 C only. |  |  | **LoE 3**  **GR B** |  |  |  |
| All ablative organ surgery should be the last resort for experienced and BPS knowledgeable surgeons only. |  |  | **LoE 3**  **GR A** |  |  |  |
| **Chronic Anal Pain Syndrome** |  |  |  |  |  |  |
| Sacral neurostimulation |  |  | **LoE 3**  **GR C** |  |  |  |
| **Fibroids** |  |  |  |  |  |  |
| The management of symptomatic uterine fibroids should follow the clinical practice guidelines of the Society of Obstetricians and Gynaecologists of Canada. |  |  |  |  |  | **LoE II-3**  **GR B** |
| **Endosalpingiosis** |  |  |  |  |  |  |
| Endosalpingiosis is an incidental histologic finding and does not appear to require specific treatment |  |  |  |  |  | **LoE II-2**  **GR B** |
| **Miscellaneous** |  |  |  |  |  |  |
| Special surgical/diagnostic methods are reserved for specific  questions but should not be part of the routine diagnostic  work-up. |  |  |  | **No reference** |  |  |

**Abbreviations**

ACOG; American College of Obstetricians and Gynecologists, ASRM; American Society of Reproductive Medicine, BPS; Bladder Pain Syndrome, BSO; Bilateral salpingo-oophorectomy, CPP; Chronic Pelvic Pain, EAU; European Association of Urology, ISPOG; International Society of Psychosomatic Obstetrics and Gynecology, RCOG; Royal College of Obstetricians and Gynaecologists, SOGC; The Society of Obstetricians and Gynaecologists of Canada

**Note**

1. American College of Obstetricians and Gynecologists (ACOG) uses A-C to grade recommendations, “A based on good and consistent scientific evidence, “B” based on limited or inconsistent scientific evidence, “C” based on consensus and expert opinion. Level of evidence were reported as “I” if evidence obtained from at least one properly designed RCT, “”II-1” if evidence from well-designed controlled trials without randomisation, “II-2” if evidence from well-designed cohort or case-control studies, “II-3” if evidence from comparisons between times or places with or without the intervention, “III” opinions of respected authorities, based on clinical experience, descriptive studies, or reports or expert committees
2. European Urology Association (EUA) uses A-C to grade recommendations, “A” based on evidence from at least one RCT, “B” recommendation based on evidence from well-conducted clinical studies but without randomisation, “C” recommendation made despite the absence of clinical studies. Level of evidence described as ‘1a” evidence from meta-analysis of RCTs, “1b” evidence from at least one RCT, “2a” evidence from well-designed controlled study without randomisation, “2b” evidence from at least one other type of well-designed quasi-experimental study, “3” evidence from well-designed non-experimental studies, such as comparative studies, correlation studies and case-reports, “4” evidence from expert committee reports or opinions or clinical experience or respected authorities.
3. Royal College of Obstetricians and Gynaecologists (RCOG) uses A-D, ‘A’ as high grade of recommendation and ‘D’ as very low and “Good Practice Point” and rated as “A” : ‘1++’ if at least one meta-analysis, systematic review or randomised clinical trial, and as “1+” if a systematic review of randomised controlled trials or a body of evidence principally of “1+” studies, “B”: as body of evidence including studies rated as “2++” if systematic reviews of case control or cohort studies or cohort studies with a low risk of bias/confounding factors or extrapolated evidence from studies as “1++’” or “1+”, ‘C’: as body of evidence including studies rated as “2+” if well-designed case-control or cohort study or extrapolated evidence from studies as “2++” and “D”: as evidence level 3 if case reports or case-series or 4, if expert opinion or extrapolated evidence from studies as “2+”.
4. Society of Obstetricians and Gynaecologists of Canada (SOGC) uses A-E to grade recommendations, “A” suggests that there is good evidence to support the recommendation for a diagnostic test/ intervention /treatment and “E” as good evidence not to support the recommendation for a diagnostic test/ intervention /treatment. Level of evidence were reported as “I” if evidence obtained from at least one properly designed RCT, “”II-1” if evidence from well-designed controlled trials without randomisation, “II-2” if evidence from well-designed cohort or case-control studies, “II-3” if evidence from comparisons between times or places with or without the intervention, “III” opinions of respected authorities, based on clinical experience, descriptive studies, or reports or expert committees
