# Supplementary material for: Evaluation of clinical practice guidelines (CPG) on the management of female chronic pelvic pain (CPP) using the AGREE II instrument
Source: Int Urogynecol J. 2021 Jun 19;32(11):2899–912. doi: 10.1007/s00192-021-04848-1 (PMC8536555; doi:10.1007/s00192-021-04848-1)
Supplement: Supplementary file 3 — (DOCX 19 kb) [file 192_2021_4848_MOESM3_ESM.docx]

**Table S5. Behavioural Interventions and Alternative Therapies for Chronic Pelvic Pain with levels of evidence (LoE) and grades of recommendation (GoR)**

| **Guideline** | **ACOG ^ii)^** | **ASRM** | **EAU ^iii)^** | **ISPOG** | **RCOG ^(iii)^** | **SOGC ^(iv)^** |
| --- | --- | --- | --- | --- | --- | --- |
| **Psychological Treatments** |  |  |  |  |  |  |
| Basic psychosomatic care should be included in the treatment concept from the beginning. |  |  |  | **No evidence** |  |  |
| Psychotherapy should be reflected and possibly integrated into the treatment concept at an early stage. |  |  |  | **No evidence** |  |  |
| Consider psychological therapy in multimodal approach for BPS |  |  | **LoE 3**  **GR B** |  |  |  |
| Try psychological interventions in combination with medical and surgical treatment, or alone in CPP. |  |  | **LoE 1a**  **GR A** |  |  |  |
| Offer behavioural strategies to the patient and his/her partner to cope with sexual dysfunctions in CPP. |  |  | **LoE 2a-3**  **GR B** |  |  |  |
| Recommend psychological treatment for refractory chronic vulvar pain. |  |  | **LoE 1b**  **GR B** |  |  |  |
| Urethral pain syndrome, when patients are distressed, it is recommended to refer them for pain-relevant psychological treatment to improve function and quality of life. |  |  | **LoE 4**  **GR B** |  |  |  |
| **Physical therapies and Exercise** |  |  |  |  |  |  |
| Referral for pelvic floor physical therapy, sex therapy or cognitive behavioural therapy, alone or in combination,  is recommended to manage the myofascial and psychosocial causes and consequences of CPP and associated dyspareunia. | **No LoE reported**  **GR B** |  |  |  |  |  |
| Patients should participate in the management of CPP due to myofascial dysfunction by actively using a home stretching and exercise program |  |  |  |  |  | **LoE II-2**  **GR B** |
| Apply pelvic floor muscle treatment as first-line treatment in patients with CPP syndrome. |  |  | **LoE 2a**  **GR A** |  |  |  |
| Biofeedback treatment is recommended in patients with pelvic pain and dyssynergic defecation. |  |  | **LoE 1a**  **GR A** |  |  |  |
| In patients with an overactive pelvic floor, biofeedback is recommended as therapy adjuvant to muscle exercises. |  |  | **LoE 1a**  **GR A** |  |  |  |
| Training of the pelvic floor muscles is recommended in CPP to improve quality of life and sexual function. |  |  | **LoE 2b**  **GR B** |  |  |  |
| The limited data do not allow for the evaluation of physiotherapy in the therapy of CPP. |  |  |  | **Systematic review** |  |  |
| Consider manual and physical therapy in first  approach for BPS. |  |  | **LoE 3**  **GR B** |  |  |  |
| Consider bladder training in patients with  little pain in BPS. |  |  | **LoE 3**  **GR B** |  |  |  |
| **Dietary modifications** |  |  |  |  |  |  |
| Women with IBS should be encouraged to amend their diet to attempt to control symptoms. |  |  |  |  | **LoE 3**  **GR 3** |  |
| Consider diet avoidance of triggering substances for BPS. |  |  | **LoE 3**  **GR C** |  |  |  |
| **Complementary and alternative therapies** |  |  |  |  |  |  |
| Use alternative therapies in the treatment of chronic gynaecological pelvic pain. |  |  | **LoE 3**  **GR C** |  |  |  |
| The limited data do not allow for an evaluation of interventions such as trigger point treatment, reflexology, biofeedback, distension therapy, homeopathy and Thiele massage in the therapy of CPP. |  |  |  | **Systematic review** |  |  |
| When myofascial trigger points are found treatment by pressure or needling is recommended. |  |  | **LoE 1a**  **GR A** |  |  |  |
| The limited data do not allow for the evaluation of acupuncture in the therapy of CPP. |  |  |  | **Systematic review** |  |  |
| Based on evidence of benefit for the treatment of non-gynaecologic chronic pain, acupuncture and yoga can be considered for the management of chronic pelvic pain of musculoskeletal aetiology. | **No LoE reported**  **GR C** |  |  |  |  |  |
| Acupuncture is not recommended for BPS |  |  | **LoE 3**  **GR C** |  |  |  |
| There is no good evidence for or against the use of TENS. Data covered chronic pain not just CPP and was insufficient regarding long-term treatment effects. |  |  | **LoE 1b GR B** |  |  |  |

**Abbreviations**

ACOG; American College of Obstetricians and Gynecologists, ASRM; American Society of Reproductive Medicine, BPS; Bladder Pain Syndrome, BSO; Bilateral salpingo-oophorectomy, CPP; Chronic Pelvic Pain, EAU; European Association of Urology, ISPOG; International Society of Psychosomatic Obstetrics and Gynecology, RCOG; Royal College of Obstetricians and Gynaecologists, SOGC; The Society of Obstetricians and Gynaecologists of Canada

**Note**

1. American College of Obstetricians and Gynecologists (ACOG) uses A-C to grade recommendations, “A based on good and consistent scientific evidence, “B” based on limited or inconsistent scientific evidence, “C” based on consensus and expert opinion. Level of evidence were reported as “I” if evidence obtained from at least one properly designed RCT, “”II-1” if evidence from well-designed controlled trials without randomisation, “II-2” if evidence from well-designed cohort or case-control studies, “II-3” if evidence from comparisons between times or places with or without the intervention, “III” opinions of respected authorities, based on clinical experience, descriptive studies, or reports or expert committees
2. European Urology Association (EUA) uses A-C to grade recommendations, “A” based on evidence from at least one RCT, “B” recommendation based on evidence from well-conducted clinical studies but without randomisation, “C” recommendation made despite the absence of clinical studies. Level of evidence described as ‘1a” evidence from meta-analysis of RCTs, “1b” evidence from at least one RCT, “2a” evidence from well-designed controlled study without randomisation, “2b” evidence from at least one other type of well-designed quasi-experimental study, “3” evidence from well-designed non-experimental studies, such as comparative studies, correlation studies and case-reports, “4” evidence from expert committee reports or opinions or clinical experience or respected authorities.
3. Royal College of Obstetricians and Gynaecologists (RCOG) uses A-D, ‘A’ as high grade of recommendation and ‘D’ as very low and “Good Practice Point” and rated as “A” : ‘1++’ if at least one meta-analysis, systematic review or randomised clinical trial, and as “1+” if a systematic review of randomised controlled trials or a body of evidence principally of “1+” studies, “B”: as body of evidence including studies rated as “2++” if systematic reviews of case control or cohort studies or cohort studies with a low risk of bias/confounding factors or extrapolated evidence from studies as “1++’” or “1+”, ‘C’: as body of evidence including studies rated as “2+” if well-designed case-control or cohort study or extrapolated evidence from studies as “2++” and “D”: as evidence level 3 if case reports or case-series or 4, if expert opinion or extrapolated evidence from studies as “2+”.
4. Society of Obstetricians and Gynaecologists of Canada (SOGC) uses A-E to grade recommendations, “A” suggests that there is good evidence to support the recommendation for a diagnostic test/ intervention /treatment and “E” as good evidence not to support the recommendation for a diagnostic test/ intervention /treatment. Level of evidence were reported as “I” if evidence obtained from at least one properly designed RCT, “”II-1” if evidence from well-designed controlled trials without randomisation, “II-2” if evidence from well-designed cohort or case-control studies, “II-3” if evidence from comparisons between times or places with or without the intervention, “III” opinions of respected authorities, based on clinical experience, descriptive studies, or reports or expert committees
